# Community health workers’ knowledge and practice in relation to pre-eclampsia in Ogun State, Nigeria: an essential bridge to maternal survival

**DOI:** 10.1186/s12978-016-0218-9

**Published:** 2016-09-30

**Authors:** J. O. Sotunsa, M. Vidler, D. O. Akeju, M. O. Osiberu, E. O. Orenuga, O. T. Oladapo, R. Qureshi, D. Sawchuck, O. O. Adetoro, P. von Dadelszen, O. A. Dada, the CLIP Nigeria Feasibility Working Group

**Affiliations:** 1 Department of Obstetrics and Gynaecology, Babcock University Teaching Hospital, Ilishan-Remo, Ogun State Nigeria; 2 Department of Obstetrics and Gynaecology, and the Child and Family Research Unit, University of British Columbia, Vancouver, V5Z 4H4 Canada; 3 Department of Sociology, University of Lagos, Lagos, Nigeria; 4 Centre for Research in Reproductive Health, Sagamu, Nigeria; 5 UNDP/UNFPA/UNICEF/WHO/World Bank Special Programme of Research, Development and Research Training in Human Reproduction (HRP), Department of Reproductive Health and Research, World Health Organization, Geneva, Switzerland; 6 Division of Women and Child Health, Aga Khan University, Karachi, Pakistan; 7 Department of Research, Vancouver Island Health Authority, Victoria, V8R 1J8 Canada; 8 Department of Obstetrics and Gynaecology, Olabisi Onabanjo University, Sagamu, Nigeria; 9 Department of Obstetrics and Gynaecology, St George’s University London, London, SW17 0RE UK

**Keywords:** Community health workers, Developing countries, Maternal welfare, Pregnancy, Nigeria, Pre-eclampsia, Hypertension in pregnancy

## Abstract

**Background:**

Pre-eclampsia is a leading cause of maternal and fetal morbidity and mortality worldwide. Early detection and treatment have been instrumental in reducing case fatality in high-income countries. To achieve this in a low-income country, like Nigeria, community health workers who man primary health centres must have adequate knowledge and skills to identify and provide emergency care for women with pre-eclampsia.

This study aimed to determine community health workers’ knowledge and practice in the identification and treatment of pre-eclampsia, as they are essential providers of maternal care services in Nigeria.

**Methods:**

This study was part of a multi-country evaluation of community treatment of pre-eclampsia. Qualitative data were obtained from four Local Government Areas of Ogun State, in south western Nigeria by focus group discussions (*N* = 15) and in-depth interviews (*N* = 19). Participants included a variety of community-based health care providers - traditional birth attendants, community health extension workers, nurses and midwives, chief nursing officers, medical officers – and health administrators. Data were transcribed and validated with field notes and analysed with NVivo 10.0.

**Results:**

Community-based health care providers proved to be aware that pre-eclampsia was due to the development of hypertension and proteinuria in pregnant women. They had a good understanding of the features of the condition and were capable of identifying women at risk, initiating care, and referring women with this condition. However, some were not comfortable managing the condition because of the limitation in their ‘Standing Order’; these guidelines do not explicitly authorize community health extension workers to treat pre-eclampsia in the community.

**Conclusion:**

Community-based health care providers were capable of identifying and initiating appropriate care for women with pre-eclampsia. These competencies combined with training and equipment availability could improve maternal health in the rural areas. There is a need for regular training and retraining to enable successful task-sharing with these cadres.

**Trial registration:**

NCT01911494.

**Electronic supplementary material:**

The online version of this article (doi:10.1186/s12978-016-0218-9) contains supplementary material, which is available to authorized users.

## Plain English summary

Pre-eclampsia, a condition characterized by elevated blood pressure and the presence of protein in the urine, is a leading cause of death in pregnancy. The risks associated with pre-eclampsia are high in low-income countries due to delays in identification, transport, and access to appropriate treatment. Many pregnant women in Nigeria live in rural areas where skilled health workers; therefore, care is often provided by minimally trained community-based health workers. As community health workers are the most available cadre of health workers, they are well positioned for early detection and initiation of care of women with pre-eclampsia from the community.

This study was conducted to determine the knowledge and practice of community health workers regarding the condition of pre-eclampsia.

To best ascertain the knowledge and practice of community health workers, fifteen focus groups and nineteen interviews were conducted. Focus groups and interviews were held with a variety of community and facility-based health workers and administrators.

This study found that overall community health workers recognize that pre-eclampsia is due to hypertension and protein in the urine. They are also aware of the features of pre-eclampsia. Nevertheless, some health workers were not comfortable treating women with pre-eclampsia. There was significant concern that treatment of pre-eclampsia was not covered in the ‘Standing Order’ guiding their practice.

With adequate training, community health workers could identify women with pre-eclampsia and may be able to provide lifesaving emergency treatment in resource-constrained areas.

## Background

Hypertension in pregnancy is a major cause of maternal mortality, with a prevalence of 1.8 to 16.7 % in low and middle-income countries (LMIC) [1]. The hypertensive disorders of pregnancy (HDP) include pre-eclampsia, gestational hypertension, chronic hypertension and superimposed pre-eclampsia [1, 2]. Pre-eclampsia is particularly challenging to manage, as there is no reliable method of detection based on clinical assessment or biochemical markers [3, 4]. In Northern Nigeria, where the maternal mortality ratio (MMR) is estimated at 1000/100,000 live births, 40 % of the deaths are due to eclampsia, a complication of pre-eclampsia [5]. In Lagos, Akinola et al. [6] reported maternal case fatality rate of 18.3 % and a perinatal mortality ratio of 279/1000 live births. In contrast, there has been a 90 % reduction in uncontrolled pre-eclampsia and eclampsia case fatality in high-income countries (HIC); this success was achieved through early detection and improved access to hospital-based care [7]. Therefore, overcoming the prevailing challenges in treating pre-eclampsia in LMIC hinges on the ability of the health care systems to identify and manage women at high-risk [1].

In resource poor settings, the unavailability of an adequate and effective health system necessitates task-sharing in the midst of the prevailing scarcity of not only funds, but also of high calibre manpower. Efforts to reduce the HDP in LMIC have been inhibited by the perceived poor quality of care, the attitude of health service providers [8, 9], and a lack of compliance with referral processes [10]. Thus, it is expedient to assess the knowledge and practice of community-based health care providers as these factors affect the quality of care delivered, and may influence acceptance of treatment and referral in the community.

Most studies on pre-eclampsia and eclampsia have assessed the causative factors, prevention and treatment without much attention to the important role of community-based providers. Factors that may contribute to high incidences of pre-eclampsia and eclampsia in LMIC include poor health seeking behaviours, and unavailability of health care facilities or qualified health personnel [8]. Therefore, community-based health care providers’ knowledge and practice is crucial.

This study aims to identify the level of knowledge of community-based health care providers and their regular practice for detection and management of pre-eclampsia. This analysis is part of a larger study looking at health system infrastructure, community and individual barriers and facilitators to maternal care services within the context of high maternal and perinatal morbidity and mortality [11].

## Methods

This was part of the feasibility study for the Community Level Intervention for Pre-eclampsia (CLIP) cluster randomized controlled trial in Ogun State, Nigeria (NCT01911494).

### Study population

The qualitative exploratory study was a conducted in four Local Government Areas (LGA) of Ogun State, Nigeria: Yewa South, Remo North, Sagamu (Ogijo-Axis), and Imeko Afon. An ethnographic approach was utilized to identify similarities and differences between groups. Where feasible, focus groups were convened; however, some groups were not possible to gather in large numbers, in these cases in-depth interviews were conducted (Tables 1 and 2). These participants represent supervisors and managerial positions, of which there are few in each LGA. Focus group discussions (FGD) (*N* = 15) and in-depth interviews (IDI) (*N* = 19) were conducted in each LGA. Focus group participants included traditional birth attendants (TBA) (*n* = 36), Community Health Extension Workers (CHEW) (*n* = 83), nurses and midwives (*n* = 43), and representatives of the Society of Obstetricians and Gynaecologists (*n* = 9). Interviews were held with the Head of the LGA administration (HOLGA) (*n* = 4), the head of the TBAs (*n* = 4), the head of the CHEWs (*n* = 4), the Chief Nursing Officer (CNO) (*n* = 4), medical officers of health (MOH) (*n* = 2), and one TBA (*n* = 1).

**Table 1:** Focus group participants

#	Participant group	Local government area	Number of participants
1	Traditional Birth Attendants	Yewa South	12
2	Traditional Birth Attendants	Remo North	12
3	Traditional Birth Attendants	Sagamu (Ogijo Axis)	12
4	Community Health Extension Workers	Sagamu (Ogijo Axis)	12
5	Community Health Extension Workers	Sagamu (Ogijo Axis)	12
6	Community Health Extension Workers	Yewa South	12
7	Community Health Extension Workers	Yewa South	12
8	Community Health Extension Workers	Imeko-Afon	12
9	Community Health Extension Workers	Imeko-Afon	12
10	Community Health Extension Workers	Remo North	11
11	Nurses and Midwives	Yewa South	12
12	Nurses and Midwives	Remo North	9
13	Nurses and Midwives	Sagamu (Ogijo Axis)	10
14	Nurses and Midwives	Imeko-Afon	12
15	Members of the Society of Obstetricians and Gynaecologists	Nigeria	9

**Table 2:** In-depth interview participants

#	Participant group	Local government area
1	Head of Local Government Administration	Sagamu (Ogijo Axis)
2	Head of Local Government Administration	Yewa South
3	Head of Local Government Administration	Imeko-Afon
4	Head of Local Government Administration	Remo North
5	Chief Nursing Officer	Remo North
6	Chief Nursing Officer	Sagamu (Ogijo Axis)
7	Chief Nursing Officer	Yewa South
8	Chief Nursing Officer	Imeko-Afon
9	Medical Officer of Health	Yewa South
10	Medical Officer of Health	Remo North
11	Head Community Health Extension Worker	Yewa South
12	Head Community Health Extension Worker	Imeko-Afon
13	Head Community Health Extension Worker	Sagamu (Ogijo Axis)
14	Head Community Health Extension Worker	Remo North
15	Head Traditional Birth Attendant	Yewa South
16	Head Traditional Birth Attendant	Imeko-Afon
17	Head Traditional Birth Attendant	Sagamu (Ogijo Axis)
18	Head Traditional Birth Attendant	Remo North
19	Traditional Birth Attendant	Imeko-Afon

A convenience sample was used. FGDs and IDIs were conducted in central community locations, to ease access for all participants. All sessions were audio-recorded and transcribed verbatim by the field researchers in the original language (Yoruba) using a structured transcription format. Transcriptions were validated by observations and review of field notes. All transcripts underwent another round of consistency checks by moderators. The validated data was then translated to English and analysed using NVivo 10.0.

Ethical approval for the study was obtained from the Olabisi Onabanjo University Teaching Hospital, Sagamu Nigeria (OOUTH/DA/326/431), and University of British Columbia, Vancouver, Canada (H12-00132).

## Results

### Knowledge of pre-eclampsia

Knowledge regarding the cause of pre-eclampsia among community-based health care providers varied. Most responses stemmed from focus groups, which stimulated discussion on the interplay of many causal factors, rather than one-on-one interviews that provided more direct and limited responses. As expected CHEWs, nurses, midwives and TBAs explained causes of pre-eclampsia to be medical in nature. Health workers, particularly nurses and midwives, stated that hypertension in pregnancy is hereditary. This finding is corroborated by beliefs related to the aetiology of pre-eclampsia expressed by the community [12]. The most consistently stated cause of pre-eclampsia by participants was psychological, with the clear majority pointing to depressive thoughts. The root of these depressive thoughts was described as due to marital conflict and/or financial worries. Problems with the husband and within the home included abandonment in pregnancy, unfaithful partners and lack of adequate care by the husband. This was captured in the statements below:

*It is caused by the husband’s bad behaviour […]; some husbands would stop taking care of their wives when they become pregnant. [Focus group discussion with community health extension workers - Imeko-Afon]*

Some nurses at primary health centres (PHC) attributed ignorance, carelessness on the part of women and their families, lack of adequate care during pregnancy and misuse of medications as contributors to hypertension in pregnancy. Again, the statements below reveal this:

*About the high blood pressure…a lot of times, it is due to ignorance […], they don’t have any knowledge of what you are talking about so it doesn’t allow them to take the situation seriously and seek treatment. [Focus group discussion with nurses and midwives - Yewa South]*

### Identification of pre-eclampsia

It was found that CHEWs were confident in their ability to accurately measure blood pressure and detect hypertension in women at the PHC, when equipment was available. It was mentioned in all eight focus groups with CHEWs and all four interviews with Head CHEWs that they are currently measuring blood pressure as part of regular practice. CHEWs, as well as their supervisors, described confidence in their ability to take blood pressure. In fact they believe “*even the junior CHEW, the least among the rank of CHEWs, […] knows how to measure a patient’s blood pressure accurately” [Focus group discussion with Head community health extension workers].*

Faced with the challenges of equipment availability, CHEWs relied heavily on observed symptoms associated with pre-eclampsia to diagnose and treat. The symptoms that were regularly reported as related to pre-eclampsia were headache, oedema, lack of sleep, and dizziness. One CHEW in Imeko-Afon described the significance of swelling as a warning signal:

*During the antenatal clinic, when we monitor the blood pressure, we monitor how the woman is doing, their legs, their hands, whether the hands and the legs are swollen, when the hands and the legs are swollen, we would refer because during their labour they develop eclamptic convulsions, so we should monitor that one so that it doesn’t happen during their labour. [Focus group discussion with community health extension workers – Imeko-Afon]*

In cases where pre-eclampsia or related symptoms were identified by CHEWs in the home or PHC, some responded by immediate referral while others continued community-based monitoring. There was not a uniform response for cases of pre-eclampsia, this likely reflects the variability in equipment and staff availability as well as their comfort level in managing such cases.

### Treatment of pre-eclampsia

The ability of CHEWs to provide emergency management for pre-eclampsia and eclampsia was explored through focus group discussions. According to international guidelines, treatment of pre-eclampsia should include the administration of oral antihypertensives for hypertension and magnesium sulphate (MgSO_4_) for the prevention and treatment of seizures [13].

It was not uniformly stated that CHEWs currently provide antihypertensive treatment for hypertension in pregnancy. Most CHEWs who claimed to provide antihypertensives, used amiloride and hydrochlorothiazide or nifedipine, while nurses listed methyldopa, paracetamol, and hydralazine with methyldopa as the most common drug. Participants described that antihypertensives were given when blood pressure was elevated under the directive of a doctor or nurse. Concern was expressed regarding whether this was allowed within the current scope of practice for CHEWs [14]. This fear may have held respondents back during the focus group discussion to freely disclose all treatments they provide.

*There are professional bodies that have been trained to carry out this but as far as Community Health Extension Workers is concerned, we are not empowered to do it. [Focus group discussion with community health extension workers – Sagamu (Ogijo-Axis)]*

The responses of CHEWs were split regarding familiarity with MgSO_4_: some claimed to know nothing about the medication, some had heard of its use, some knew of it in detail, yet were not using it, and finally some were using it regularly. One CHEW from Sagamu (Ogijo-Axis) explained her knowledge of the medication:

*They […] give it to those with high blood pressure. It is not in our clinic but I know about it. It is in a powder form that can be diluted, it is for control of blood pressure so that it doesn’t lead to convulsion or to prevent eclampsia, it is an injection that is given through the intravenous or intramuscular route. [Focus group discussion with community health extension workers – Sagamu (Ogijo-Axis)]*

Doctors and nurses, not surprisingly, were most familiar with MgSO_4._ Some were using it regularly while others continued not to use it in cases of pre-eclampsia or eclampsia. One of the explanations for the lack of its use was attributed to variable procurement of MgSO_4_ in facilities. Although some health care providers were familiar with MgSO_4_, most were not well versed in its use_;_ in these cases other anticonvulsant medications were regularly provided. It was repeatedly stated by CHEWs that diazepam was the treatment of choice in pre-eclampsia. Similarly nurses, midwives and faith based providers stated using diazepam and referring in cases of hypertension. There were some acknowledgements of the risks associated with diazepam; however, it was used in spite of these concerns. The confidence of CHEWs in the administration of MgSO_4_ yielded divergent responses. The level of comfort in the use of the drug was influenced by both experience and training.

Overall, CHEWs claimed to be comfortable managing pre-eclampsia and referring when necessary. This is demonstrated by the statement below:

*If they have high blood pressure, if it’s something that we can still manage, they will accept the treatment, otherwise anywhere you refer them to is where they will go. [Focus group discussion with community health extension workers – Imeko-Afon]*

CHEW supervisors stated that additional training is required for CHEWs to effectively identify and manage women suffering from pre-eclampsia. This training should include identification of warning signals and medication administration.

*All the CHEWs in this Local Government need to be trained on how to identify pregnant women with high blood pressure. Gather them; design a programme for them on how to identify danger signs of pregnancy. If that one is done, I believe it will go a long way to improve the health of the pregnant women.*

*They would need training on this. You can invite a pharmacist to come and train them on the dosage and how many milligrams of the drugs, so that they will not overstep their boundaries. [Interview with the head community health extension worker – Imeko-Afon]*

Representatives of the Society of Obstetricians and Gynaecologists of Nigeria (SOGON) argued that the training of CHEWs should be comprehensive and include identification, triage and treatment for women with pre-eclampsia and eclampsia. The suggested training should focus predominantly on clinical skills.

*[CHEWs] must be able to recognize signs and symptoms of pre-eclampsia and eclampsia and they must also be trained to be able to do the blood pressure and also check the urine for urinalysis, there are so many dipsticks easy for anybody to use now. They must be trained properly and there must be adequate monitoring of these health extension workers. You must adequately monitor them and also train and retrain them. You don’t just leave them alone to do whatever they like. [Focus group discussion with representatives of the Society of Obstetricians and Gynaecologists of Nigeria]*

## Discussion

### Principal findings

Communities commonly believe that spiritual attacks, carelessness of the woman and neglect from her husband are causes of the hypertensive disorders of pregnancy [12, 15]. Despite these pervasive community misconceptions, community-based health care providers were aware of pre-eclampsia and its features. This study showed that these providers in Ogun State have some knowledge of pre-eclampsia as well as its complications. They were capable of identifying related symptoms, initiating care and referring women when appropriate. However, the fact that many in the community still have misconceptions regarding pre-eclampsia reinforces the need for “*intensive education of the pregnant women by the health worker*”, as stated by James et al. [12, 16]. This underscores the need for consistent knowledge amongst the community providers so as to guarantee increased education and skills for all.

### Relation to other findings

It is important to note the hesitation of many to treat pre-eclampsia, often due to the uncertainty of the standing order [14]. As noticed in Pakistan by Bigdeli et al., there was a similar reluctance among this cadre due to restrictions imposed by guidelines [17]. This calls for improved communication of the guidelines, particularly in regions where there is increasing initiatives to redistribute health care responsibilities through task-sharing.

This study, as that of Kim et al. [3] in Afghanistan, found that there is sub-optimal knowledge regarding the usage of MgSO_4_ among community health workers. However, others have demonstrated that low cadre health workers at PHCs can safely administer MgSO_4_ women with severe pre-eclampsia and eclampsia [10]. CHWs had limited exposure to MgSO_4_; however, they were very familiar with injections of other types, particularly immunizations.

### Implication of this study

Existing community health worker trainings provided by the government are neither comprehensive nor sufficient for the required skills to appropriately manage pregnancy complications; they, therefore, must be supplemented. Comprehensive training should encompass domiciliary visits, identification of pregnancy complications and the safe administration of oral medications and injectables in pregnancy. One-time schooling is insufficient and refresher courses should be provided to regularly reinforce these skills to community health workers.

### Strengths and limitations

The qualitative nature of this study allowed participants to express themselves freely without inhibition. The possibility of bridging the manpower deficit through task sharing with these category of health care workers is a reality. The study however does not show how these can be sustained.

Though participants were aware of pre-eclampsia, the specifics of appropriate treatment were often unknown and confidence is lacking in their ability to manage such complications. Enhanced skills of community-based providers would benefit the community and health system as a strategy for closing the gap of services due to human resource constraints in Nigeria. Further research is required to find ways of sustaining the knowledge and the use of such knowledge to limit the menace of pre-eclampsia in the community.

## Conclusion

Community health workers had at least basic knowledge of pre-eclampsia; however, their confidence in the detection and appropriate management mandates further training and retraining coupled with adequate understanding of their role in the overall health care system, especially in the rural areas.

## Additional file

Additional file 1: Reviewer reports. (PDF 359 kb)

## Abbreviations

CHEW: Community health extension workers
CHW: Community health worker
CLIP: Community level interventions for pre-eclampsia
CNO: Chief nursing officer
FGD: Focus group discussion
HDP: Hypertensive disorders of pregnancy
HIC: High-income countries
HOLGA: Head of LGA administration
IDI: In-depth interview
LGA: Local government areas
LMIC: Low and middle-income countries
MMR: Maternal mortality ratio
MOH: Medical officer of health
PHC: Primary health centre
SOGON: Society of Obstetricians and Gynaecologists of Nigeria
TBA: Traditional birth attendants
