# Supplementary material for: Community health workers’ knowledge and practice in relation to pre-eclampsia in Ogun State, Nigeria: an essential bridge to maternal survival
Source: Reprod Health. 2016 Sep 30;13(Suppl 2):108. doi: 10.1186/s12978-016-0218-9 (PMC5056496; doi:10.1186/s12978-016-0218-9)
Supplement: Additional file 1: — Reviewer reports. (PDF 359 kb) [file 12978_2016_218_MOESM1_ESM.pdf]

Referee's comments to the authors– this sheet WILL be seen by the author(s) and published with the article

Title	Community health workers' knowledge and practice in relation to pre-eclampsia in Ogun State, Nigeria: An essential bridge to maternal survival
Author(s)	Sotunsa, JO, Vidler, M, Akeju, DO, Osiberu, MO, Orenuga, EO, Oladapo, OT, Qureshi, R, Sawchuck, D, Adetoro, OO, von Dadelszen, P, Dada, O and the CLIP Nigeria Feasibility Working Group
Referee's name	Rosnah Sutan

When assessing the work, please consider the following points, where applicable:

1. Is the question posed by the authors new and well defined?
2. Are the methods appropriate and well described, and are sufficient details provided to replicate the work?
3. Are the data sound and well controlled?
4. Does the manuscript adhere to the relevant standards for reporting and data deposition?
5. Are the discussion and conclusions well balanced and adequately supported by the data?
6. Do the title and abstract accurately convey what has been found?
7. Is the writing acceptable?

Please make your report as constructive and detailed as possible in your comments so that authors have the opportunity to overcome any serious deficiencies that you find and please also divide your comments into the following categories:

- Major Compulsory Revisions (which the author must respond to before a decision on publication can be reached)
- Minor Essential Revisions (such as missing labels on figures, or the wrong use of a term, which the author can be trusted to correct)
- Discretionary Revisions (which are recommendations for improvement but which the author can choose to ignore)

Where possible please supply references to substantiate your comments.

When referring to the manuscript please provide specific page and paragraph citations where appropriate.

General comments:

In overall it is a good study to eplore CHW knowledge and practice at their coverage areas. However, proper methodology documentation would better explain the aims why the need of such study.

Major compulsory revisions:
Line 42: the aims of the study to determine community health knowledge and competence? Why it is different from the title? Practice or competence?
Line 46: state type of study design you use under qualitative method.
Line 91: "….to assess knowledge and practice…" which one practice or competence?
Line 99**:** "This study aims to identify the level of knowledge of CHWand their regular practice.." please be consistent which one competence or practice? Why you need to repeat your aim of the study again?
Line 105: please elaborate more on current study that is written in this manuscript: qualitative study design? Approach? Sampling technique? Sample unit? Tools use (key questions asked). How the FGD been conducted, how many in a group? Homogenousity of the respondents?

:

(continue on the next sheet)

Table 1& 2 shows the distribution of the respondents. Why you need to do in depth interview and FGD? What is the purpose using 2 different techniques from same category of CHW? What are the key questions?

It is clearer if the type of CHW minimum competency skills to handle pregnant cases is stated.
Line 182: write MGSO4 in full then bracket with initial.

Line 184: "Most CHEWs who claimed to provideantihypertensives,used 184 amiloride and hydrochlorothiazide or nifedipine, while nurses listed methyldopa, paracetamol, 185 and hydralazinewith methyldopaas the most common drugs." Are only these drugs are available based on the guideline practice in this place? are the drugs have enough supply?

Referee's comments to the authors– this sheet <u>WILL</u> be seen by the author(s) and published with the article

Title	Community health workers' knowledge and practice in relation to pre-eclampsia in Ogun State, Nigeria: An essential bridge to maternal survival
Author(s)	Sotunsa, JO, Vidler, M, Akeju, DO, Osiberu, MO, Orenuga, EO, Oladapo, OT, Qureshi, R, Sawchuck, D, Adetoro, OO, von Dadelszen, P, Dada, O and the CLIP Nigeria Feasibility Working Group
Referee's name	Gita Dhakal

When assessing the work, please consider the following points, where applicable:

1. Is the question posed by the authors new and well defined?
2. Are the methods appropriate and well described, and are sufficient details provided to replicate the work?
3. Are the data sound and well controlled?
4. Does the manuscript adhere to the relevant standards for reporting and data deposition?
5. Are the discussion and conclusions well balanced and adequately supported by the data?
6. Do the title and abstract accurately convey what has been found?
7. Is the writing acceptable?

Please make your report as constructive and detailed as possible in your comments so that authors have the opportunity to overcome any serious deficiencies that you find and please also divide your comments into the following categories:

- Major Compulsory Revisions (which the author must respond to before a decision on publication can be reached)
- Minor Essential Revisions (such as missing labels on figures, or the wrong use of a term, which the author can be trusted to correct)
- Discretionary Revisions (which are recommendations for improvement but which the author can choose to ignore)

Where possible please supply references to substantiate your comments.

When referring to the manuscript please provide specific page and paragraph citations where appropriate.

General comments:
No revisions needed

Major compulsory revisions:

Minor essential revisions:

Discretionary revisions:

(continue on the next sheet)

In addition the editor is asking for major changes particularly in the Discussion section.
The Discussion section is not well organized. Please try to better organize this section. The first sentences of this section are out of the findings of the study. It would be good if you can follow the following scheme for this section:

* statement of principal findings of the study. Summarise key results with reference to study objectives
* strengths and weaknesses of the study
* strengths and weaknesses in relation to other studies, discussing important differences in results and what your study adds. Whenever possible please discuss your study in the light of relevant systematic reviews and meta-analyses (eg Cochrane reviews)
* meaning of the study: possible explanations and implications for clinicians and policymakers and other researchers; how your study could promote better decisions
* unanswered questions and future research

Lines 59-60. It is not clear if it is a finding or a recommendation derived from the study. We are promoting the use of the term sharing instead of shifting.
